# Logic Regression for Provider Effects on Kidney Cancer Treatment Delivery

**DOI:** 10.1155/2014/316935

**Published:** 2014-03-27

**Authors:** Mousumi Banerjee, Christopher Filson, Rong Xia, David C. Miller

**Affiliations:** ^1^Department of Biostatistics, School of Public Health, University of Michigan, 1415 Washington Heights, Ann Arbor, MI 48109, USA; ^2^Center for Healthcare Outcomes & Policy, University of Michigan, 2800 Plymouth Road, Ann Arbor, MI 48105, USA; ^3^Department of Urology, University of Michigan, 1500 E Medical Center Drive, Ann Arbor, MI 48109, USA

## Abstract

In the delivery of medical and surgical care, often times complex interactions between patient, physician, and hospital factors influence practice patterns. This paper presents a novel application of logic regression in the context of kidney cancer treatment delivery. Using linked data from the National Cancer Institute's (NCI) Surveillance, Epidemiology, and End Results (SEER) program and Medicare we identified patients diagnosed with kidney cancer from 1995 to 2005. The primary endpoints in the study were use of innovative treatment modalities, namely, partial nephrectomy and laparoscopy. Logic regression allowed us to uncover the interplay between patient, provider, and practice environment variables, which would not be possible using standard regression approaches. We found that surgeons who graduated in or prior to 1980 despite having some academic affiliation, low volume surgeons in a non-NCI hospital, or surgeons in rural environment were significantly less likely to use laparoscopy. Surgeons with major academic affiliation and practising in HMO, hospital, or medical school based setting were significantly more likely to use partial nephrectomy. Results from our study can show efforts towards dismantling the barriers to adoption of innovative treatment modalities, ultimately improving the quality of care provided to patients with kidney cancer.

## 1. Introduction 

Open radical nephrectomy has long been the standard treatment for patients with early-stage kidney cancer [1]. In recent years, however, easier convalescence and equivalent cancer control established laparoscopy as an alternative standard of care for most patients treated with radical nephrectomy [1–3]. Studies have also demonstrated that, for patients with small renal masses, partial instead of radical nephrectomy achieves identical cancer control while better preserving long-term renal function and reducing overtreatment of benign or clinically indolent tumors [4–7]. However, despite their potential advantages, the adoption of laparoscopy and partial nephrectomy have been relatively slow and asymmetric in the population [3, 8].

Earlier studies have shown that individual surgeon characteristics and their practice environments largely influence the use of laparoscopy and partial nephrectomy [9]. These studies are based on logistic regression models, a member of the generalized linear model family suitable for data with a binary outcome (e.g., use versus nonuse of laparoscopy). Logistic regression focuses on identification of* main effects*. While interactions can be assessed using logistic regression, these interactions need to be known a priori and specified as input variables in the model.* Discovery of interactions* is therefore difficult using logistic regression. We hypothesize that surgeon characteristics may not have uniform effect on the adoption of laparoscopy and partial nephrectomy across practice environments. For example, use of advanced techniques may vary among recently trained surgeons depending on the surgeon's affiliation with an academic hospital or NCI-designated cancer center, suggesting a potential interaction between year of medical school graduation and practice setting.

Logic regression is an adaptive classification and regression procedure [10], initially developed to uncover and measure the importance of interacting factors in genetic association studies [11, 12]. There are many approaches based on classification methods such as CART and Random Forests [13–15] that allow measuring the importance of a single predictor. But none of these methods can directly quantify the importance of combinations of several predictors. Logic regression uses the predictors as inputs into the model while still enabling one to identify combinations of predictors and quantify the importance of these interactions.

In general, logic regression can be used in any setting, when the interaction between the predictors is of primary interest. Logic regression searches for Boolean (logical) combinations of the original predictors that best explain the variability in the outcome variable and, thus, reveals variables and interactions that are associated with the response and/or have predictive capabilities. Given a set of binary predictors, one creates new predictors such as “*X*_1_, *X*_2_, *X*_3_, and *X*_4_ are true” or “*X*_5_ or *X*_6_ but not *X*_7_ is true.” In more specific terms, the goal is to try to fit regression models of the form logit[*P*(*Y* = 1)] = *b*_0_ + *b*_1_ *L*_1_ + ⋯+*b*_*p*_ *L*_*p*_, where *P*(*Y* = 1) is the probability that the binary outcome is 1, and *L*_*j*_ is any Boolean expression of the predictors. The *L*_*j*_ and *b*_*j*_ are estimated simultaneously using a stochastic optimization algorithm [16].

The goal of this paper is to introduce logic regression as a novel method for discovering interactions, specifically, Boolean combinations of factors that potentially discriminate users of partial nephrectomy or laparoscopy from nonusers. Characterizing providers who are actually using (or not using) these techniques is needed to show education- and/or policy-based interventions designed to increase utilization of these advanced surgical techniques. Given that logic expressions are embedded in a generalized linear regression framework and therefore naturally adaptable to other outcome types (e.g., numeric and time-to-event data); the method has broad scope of application in health services and outcomes research.

## 2. Materials and Methods 

### 2.1. Data Source

We used data from the National Cancer Institute's Surveillance, Epidemiology, and End Results (SEER) Program and the Centers for Medicare and Medicaid Services (Medicare) to identify patients diagnosed with incident kidney cancer from 1995 to 2005. SEER is a population-based cancer registry that collects data regarding incidence, treatment, and mortality. The demographic composition, cancer incidence, and mortality trends in the SEER registries are representative of the entire United States population. The Medicare Program provides primary health insurance for 97% of the United States population aged 65 years and older, and linkage to Medicare claims is achieved for >90% of SEER cases over age 65 [17].

### 2.2. Cohort Identification and Assignment of Surgical Procedure

We identified 15,744 patients diagnosed with nonurothelial, nonmetastatic kidney cancer from 1995 to 2005. For this group of patients, we searched inpatient and physician claims to identify kidney cancer-specific diagnosis and procedure codes (list of codes available from authors upon request). We excluded patients who lacked claims denoting surgical treatment for kidney cancer, patients with multiple hospitalizations for direct open or partial nephrectomy, patients whose claims suggested the presence of bilateral tumors at diagnosis, and patients operated by a nonurologic specialty physician. This process yielded a cohort of 11,918 cases. We applied a validated claim-based algorithm to assign each patient to one of four mutually exclusive surgical categories: (1) open radical nephrectomy (ORN) (*n* = 8029), (2) open partial nephrectomy (OPN) (*n* = 1380), (3) laparoscopic radical nephrectomy (LRN) (*n* = 2082), and (4) laparoscopic partial nephrectomy (LPN) (*n* = 427).

As validation, we assessed the level of concordance between our claim-based algorithm and the type of cancer-directed surgery specified for each patient in the SEER data file (Patient Entitlement and Diagnosis Summary File). Although SEER does not collect data regarding whether the surgical approach was open or laparoscopic, we observed 97% agreement for the assignment of partial versus radical nephrectomy (*κ* = 0.83). Also, we identified relevant surgical pathology claims within 30 days of the index admission for more than 95% of analyzed cases, thus supporting the occurrence of cancer-directed surgery. As a final step, we externally validated our algorithm by comparing procedure assignments based on Medicare claims with the surgery specified in actual operative reports of 549 cases from the Los Angeles Cancer Surveillance Program. Overall, the claims-based algorithm assigned the correct surgical procedure (ORN, OPN, LRN, or LPN) for 97% of patients in the validation sample (*κ* = 0.91). We observed equally high concordance for identification of laparoscopic versus open surgery (*κ* = 0.87) and for classification of partial versus radical nephrectomy (*κ* = 0.93).

### 2.3. Patient-Level Covariates

For each patient in the analytic cohort, we used SEER data to determine demographic and cancer-specific information (i.e., age at surgery, gender, race/ethnicity, marital status, tumor size, tumor grade, histology, and laterality). Based on patient-level zip codes, we assigned patients to one of three socioeconomic strata [18]. We measured preexisting comorbidity by using a modified Charlson Index based on claims submitted during the 12 months prior to the kidney cancer surgery [19, 20].

### 2.4. Primary Surgeon and Surgeon-Level Covariates

To identify the primary surgeon for each case, we used encrypted Unique Physician Identifier Numbers (UPIN) submitted with Medicare physician claims. We linked the comprehensive list of surgeon UPINs to the American Medical Association (AMA) Physician Masterfile, which contains demographic, educational, and certification information for over one million residents and physicians in the United States. Using AMA data, we determined surgeon age, gender, year of medical school graduation, and practice size. We assigned each surgeon a rural-urban commuting area (RUCA) code based on an established classification scheme using the zip code of the primary office address [21]. We determined academic affiliation (major, minor, or no academic affiliation) based on the methods described by Shahinian et al. [22]. We also determined each surgeon's average annual nephrectomy (partial or radical) volume using claims from 1995 to 2005. We empirically defined high-volume surgeons as those performing at least 3 annual cancer-related nephrectomies among the SEER-Medicare population (83rd percentile). This measure of case volume may not reflect the total number of nephrectomies performed by a provider: it fails to account for surgeries among younger (non-Medicare-eligible) patients, Medicare HMO enrollees, and/or fee-for-service Medicare participants who reside outside of the SEER registries. Finally, we determined each surgeon's association with a National Cancer Institute- (NCI-) designated cancer center based on whether or not they performed at least one radical nephrectomy at a hospital carrying this designation.

### 2.5. Statistical Methods

Before fitting logic regression models, we performed several univariate analyses. We used Chi-square tests to evaluate the level of association between surgical procedure and various patient-level covariates and to assess the statistical significance of temporal surgical trends. For the subsequent modeling, we defined two binary endpoints as follows: (1) use of partial nephrectomy (i.e*.,* OPN+LPN versus ORN+LRN) and (2) use of laparoscopy among patients who underwent radical nephrectomy (i.e., LRN versus ORN).

The classification algorithm used in this study is logic regression, an adaptive regression methodology developed by Ruczinski et al. [10]. In the logic regression framework, given a set of binary covariates *X*, the goal is to create new, better predictors for the response by constructing Boolean combinations of the binary covariates. For example, if the response is binary, the goal is to find decision rules such as “if *X*_1_, *X*_2_, *X*_3_, and *X*_4_ are true,” or “*X*_5_ or *X*_6_ but not *X*_7_ is true,” then the response is more likely to be in class 0. Boolean combinations of the covariates, called logic trees, are represented graphically as a set of and-or rules. Logic regression searches for Boolean combinations of predictors in the entire space of such combinations, while being completely embedded in a regression framework, where the quality of the models is determined by an appropriate score function for the regression class.

Let *X*_1_, *X*_2_,…, *X*_*k*_ be binary (0/1) predictors and let *Y* be the response. In our setting, *X*'s correspond to patient, physician, and practice environment variables, and *Y* represents binary outcomes (use of partial nephrectomy: yes/no; use of laparoscopy: yes/no) each of which is modeled separately using binomial deviance as the score function. For a given set of Boolean expressions, an example of which was given in Section 1, the logic regression model is a logistic regression model with those Boolean expressions as covariates. Specifically, we denote a Boolean expression with the binary variable *L*, where *L* = 1 is true and *L* = 0 is false. The model is written as

(1) $$\begin{array}{c} \text{logit}P\left(Y=1\;|\;L_{1},\ldots ,L_{p}\right)=\beta_{0}+\beta_{1}L_{1}+\cdots +\beta_{p}L_{p}, \end{array}$$

where *L*_*j*_ is a Boolean combination of the predictors *X*_*i*_'s. The goal is to find Boolean expressions in (1) that minimize the binomial deviance, estimating the parameters *β*_*j*_ simultaneously with the search for the Boolean expressions *L*_*j*_. This is what distinguishes logic regression from simple logistic regression with binary covariates, that is, that the fitting algorithm both defines “covariates” for model (1) (using predictor data) and estimates the regression coefficients simultaneously. The output from logic regression is represented as a series of trees, one for each Boolean predictor, *L*_*j*_, and the associated regression coefficient.

CART is another tree-based method for modeling binary data [15]. The classification rule is displayed as a tree whose leaves are the two classes of interest (e.g., use versus nonuse of partial nephrectomy or laparoscopy) and whose branches correspond to dichotomized covariates. Each leaf is reached by one or more paths through the tree; to reach the leaf, all conditions along the path must be satisfied. Thus, a classification tree can be thought of as the collection of all paths that reach a leaf predicting use of treatment. Therefore, any classification tree can be written as a Boolean combination of covariates, as can a logic regression tree. However, there are some Boolean expressions which can be very simply represented as logic trees, but which require fairly complicated classification trees [10]. It is this simplicity of logic trees which we hope to exploit in order to produce easily interpretable characterizations of individuals who have a high likelihood of using the specific surgical treatment.

In logic regression, the challenge is to find good candidates for the logic term *L*_*j*_, as the collection of all Boolean expressions is enormous. Using a tree-like representation for logic expressions, we adaptively select this term using a simulated annealing algorithm [16]. In our setting leaves of each tree are the threshold conditions for each covariate, and the root and knots of the tree are the Boolean (and-or) operators. Simulated annealing is a stochastic optimization algorithm. At each step a possible operation on the current tree, such as adding or removing a knot, is proposed at random. This operation is always accepted if the new logic tree has a better score than the old logic tree; otherwise, it is accepted with a probability that depends on the difference between the scores of the old and the new tree and the stage of the algorithm. Properties of the simulated annealing algorithm depend heavily on Markov chain theory and thus on the set of operations that can be applied to logic trees.

The complexity of a logic regression model is defined by the number of logic trees (*p* in (1)) and the number of variables, or leaves, that make up a tree. As with any adaptive regression methodology, larger models (those with more trees and leaves) typically fit better than smaller models. To avoid overfitting, in this paper we chose the model size using a cross-validation approach. We varied model complexity from 1 to 4 trees (corresponding to the *p* in (1)) and the number of leaves that make up a tree from 1 to 15. We randomly divided our data into ten subsets, such that each subset consisted of one-tenth of the “treatment” and the “control” (RN for the first endpoint and ORN for the second endpoint) groups. Of the ten subsets, we used nine subsets as training data and the remaining single subset as validation data for testing the model. We used the training data to develop the logic models using simulated annealing algorithm and then estimated the deviance based on the test data. This process was repeated ten times, with each of the ten subsets used exactly once as validation data (tenfold cross validation). The results from the ten folds were then averaged to produce a single deviance score for each model. To reduce variability, this procedure (splitting the data into ten parts, developing logic rules on the training data, and estimating the deviance based on the test data) was repeated 15 times, with different random splits of the whole dataset for each run. The deviance scores were averaged over the 15 rounds of cross-validation, and the model with the smallest average deviance was selected. Results presented correspond to the run yielding value for the test set based model deviance that was closest to its average across the 15 runs. This run was selected so as to provide results for what might be considered a typical rather than an extreme split of the data into test and training sets.

Logic regression requires binary predictor variables, so we recoded variables into binary forms. Categorical covariates were coded as a set of indicator variables for each level of the covariate. For example, marital status was analyzed as married versus others, and race was coded as a set of indicator variables based on the categories Caucasian, African American, Hispanic, and others. Continuous and ordinal covariates were coded as a series of threshold indicators based on a priori knowledge about the variables. For example, tumor size was categorized into two clinically relevant groups based on a 4 cm threshold; patient's age at surgery and Charlson comorbidity index were each coded as a series of threshold indicators based on five-year age intervals 65–69, 70–74, 75–79, 80–84, and ≥85 years and 0, 1, and ≥2 comorbid conditions, respectively. Each of the surgeon variables, that is, surgeon's year of medical school graduation, age, practice structure, and academic affiliation, was also coded as a series of threshold indicators based on the categories prior to 1960, 1961–1970, 1971–1980, 1981–1990, and 1991 and after; <40, 40–49, 50–59, and ≥60 years; solo or two person practice, group practice, HMO or hospital based practice, medical school, and others; and none, minor, and major affiliation, respectively.

## 3. Results 

We identified a total of 11,918 Medicare beneficiaries who underwent surgery for an incident kidney cancer diagnosed between 1995 and 2005.  Table 1 presents demographic and clinical characteristics of patients in the analytic sample. During the study interval, 1807 patients (15.2%) underwent partial nephrectomy (427 performed laparoscopically), and 10,111 patients (84.8%) underwent radical nephrectomy (2082 performed laparoscopically). We observed differences in treatment patterns according to patient age, gender, race/ethnicity, marital status, socioeconomic status, tumor size, tumor grade, and histology (Table 1).

From 1995 to 2005, the annual proportion of patients who underwent partial nephrectomy increased from 8.5% to 21.3% (*P* < 0.0001); for patients who had tumors that measured ≤4 cm, the proportion rose from 14.4% to 37.1% (*P* < 0.0001). Among patients treated with radical nephrectomy, the annual proportion of laparoscopy use increased from 1.3% in 1995 to 44.1% in 2005 (*P* < 0.0001). For patients whose tumors measured ≤4 cm, the annual proportion of laparoscopy use increased from 1.6% to 52.9%; for patients with larger tumors, this proportion increased from 1.2% to 39.3% (*P* values <0.0001).

We identified 2088 primary surgeons who performed 11,918 kidney cancer surgeries during the study interval (median 4 cases). Of these, 2019 surgeons performed 10,111 radical nephrectomies (median 3 cases; range 1–84). During the same interval, 842 surgeons performed 1,807 partial nephrectomies (median 1 case; range 1–29). Of the 2019 surgeons who performed the radical nephrectomies, 720 operated laparoscopically on 2,082 patients (median 2 cases; range 1–55). We observed differences in treatment patterns according to provider age, gender, year of medical school graduation, annual nephrectomy volume, practice size, rural/urban status, academic affiliation, and NCI cancer center designation (Table 2).

**Figure 1:**
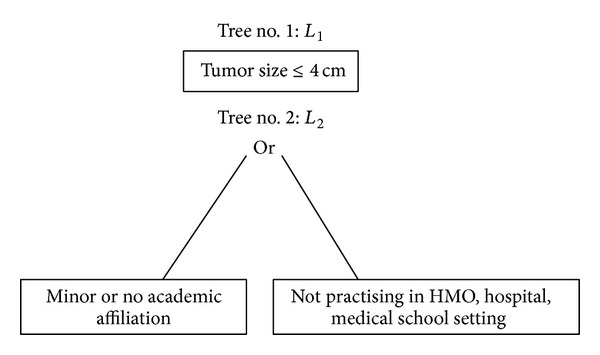
Two-tree model for use of partial nephrectomy. The odds ratio associated with *L*_1_ is 5.9 (95% CI 4.7–7.4) and that with *L*_2_ is 0.30 (95% CI 0.23–0.39).

Figure 1 displays results of the logic regression to determine optimal combination rules for use of partial nephrectomy based on a two-tree model. The first tree, *L*_1_, is entirely described by tumor size. The estimated odds ratio associated with this tree is 5.9 (95% CI 4.7–7.4), suggesting that tumor size ≤4 cm is associated with almost six times higher odds of partial nephrectomy. This finding is concurrent with previous reports in the literature documenting tumor size as a strong predictor of partial nephrectomy [8, 9]. Interestingly, the second tree, *L*_2_, involves practice environment characteristics. This tree (*L*_2_) indicates that not having major academic affiliation, or not practising in HMO, hospital, medical school based setting is associated with lower odds ratio of partial nephrectomy. The estimated odds ratio associated with *L*_2_ is 0.30 (95% CI 0.23–0.39), suggesting that, as a group, those satisfying *L*_2_ are estimated to have a 70% lower odds of using partial nephrectomy compared to those who do not satisfy the tree. In other words, patients treated by surgeons who have major academic affiliation and are in HMO, hospital, or medical school based practice setting are 3.3 times more likely to undergo partial nephrectomy than their counterparts.

**Figure 2:**
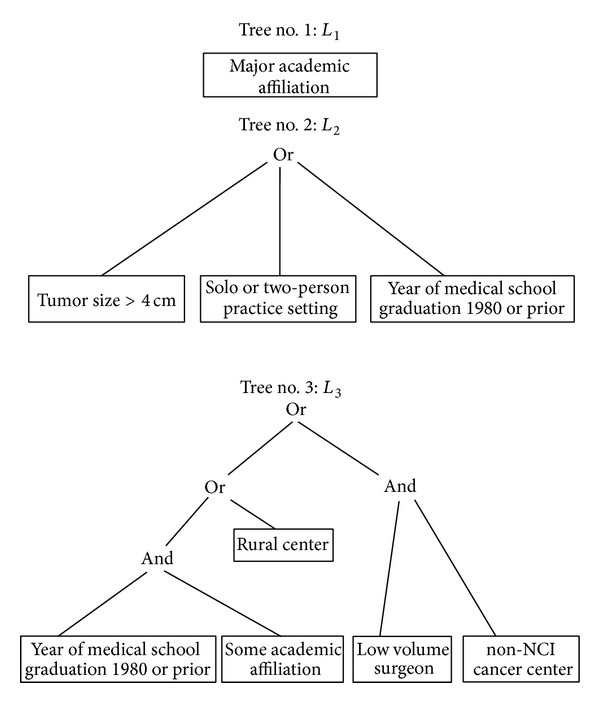
Three-tree model for use of laparoscopic radical nephrectomy. The odds ratio associated with *L*_1_ is 2.1 (95% CI 1.7–2.6), that with *L*_2_ is 0.38 (95% CI 0.29–0.48), and that with *L*_3_ is 0.29 (95% CI 0.23–0.38).

Figure 2 displays results of the logic regression to determine optimal combination rules for use of laparoscopic radical nephrectomy based on a three-tree model. The first tree, *L*_1_, is entirely described by academic affiliation. The estimated odds ratio associated with this tree is 2.12 (95% CI 1.71–2.63), suggesting that surgeon's affiliation with a major academic center is associated with two times higher odds of a laparoscopic radical nephrectomy. The second tree, *L*_2_, involves a combination of patient and surgeon characteristics. This tree (*L*_2_) indicates that having larger tumors (>4 cm) or having a surgeon who graduated in or prior to 1980 or practising in nongroup settings (solo or two person) is associated with a lower odds of laparoscopic procedure. The estimated odds ratio associated with *L*_2_ is 0.38 (95% CI 0.29–0.48), suggesting that, as a group, those satisfying *L*_2_ are estimated to have a 62% lower odds of laparoscopic radical nephrectomy compared to those who do not satisfy the tree. The third tree, *L*_3_, is characterized by a combination of surgeon and practice environment variables. This tree (*L*_3_) indicates that low volume surgeons in a non-NCI hospital, surgeons in rural environment, or surgeons who graduated in or prior to 1980 despite having some academic affiliation have a lower odds for laparoscopic procedure (odds ratio = 0.29, 95% CI 0.23–0.38).

We also performed CART analyses of our data (results not shown) for both the partial and laparoscopic radical nephrectomy endpoints. For partial nephrectomy, the CART tree yielded subgroups characterized by tumor size and surgeon's academic affiliation. As observed before, patients with tumor size > 4 cm were less likely to undergo partial nephrectomy compared to those with smaller tumors. For the latter group (tumor size ≤4 cm), surgeons with major academic affiliation had higher propensity for partial nephrectomy compared to those with minor or no academic affiliation. The area under the ROC curve for the CART tree was 0.72, compared to 0.77 for the logic regression model. For laparoscopic radical nephrectomy, the CART tree yielded subgroups characterized by surgeon's academic affiliation, year of medical school graduation, and annual surgeon volume. High volume surgeons who graduated after 1980 and were affiliated with a major academic center had the highest propensity towards laparoscopic procedure. Surgeons with minor or no academic affiliation had the lowest propensity towards laparoscopic procedure. Interestingly, despite having major academic affiliation surgeons who graduated in or prior to 1980 had only a slightly higher propensity towards laparoscopic procedure compared to surgeons with minor or no academic affiliation. The area under the ROC curve for the CART tree was 0.61, compared to 0.71 for the logic regression model.

## 4. Conclusions 

The principal finding from this study was our ability to uncover the interplay between patient, provider, and practice environment variables towards adoption of partial nephrectomy and laparoscopy. Through the use of logic regression we were able to uncover interactions that would not have been detected by standard logistic regression approach. Our findings demonstrate that the adoption of laparoscopic radical nephrectomy is particularly influenced by complex combinations of surgeon and practice environment characteristics, rather than simple “main effects.” More specifically, our results suggest that patients treated by surgeons who graduated in or prior to 1980 despite having some academic affiliation, low volume surgeons in a non-NCI hospital, or surgeons in rural environment were significantly less likely to use laparoscopic radical nephrectomy. Although less dramatic, the adoption of partial nephrectomy is also influenced by combination of tumor and practice environment characteristics. Collectively, these findings highlight the rich contextual interactions that influence urologist's adoption of new technologies and potentially reflect role of resources and access to informational externalities that help promote the adoption of these technologies.

According to Donabedian's structure-process-outcome model for quality-of-care assessment, characteristics of individual providers and their practice environments are structural measures that influence patient outcomes both directly and through their influence on specific processes of care [23]. In fact, these links have been validated empirically in multiple, diverse clinical settings. One well-characterized example is the inverse association between surgeon case volume (a provider characteristic and structural measure) and operative mortality (a patient outcome) following high-risk cancer surgery [24]. Likewise, among patients with prostate cancer, evidence-based utilization of androgen deprivation therapy (a process of care) varies based on characteristics of the treating urologist, including years since medical school graduation and academic affiliation [22]. In addition to a surgeon's individual characteristics, the practice environment also influences treatment decisions and patient outcomes. For instance, patients receiving care at the National Cancer Institute- (NCI-) designated cancer centers have lower adjusted mortality rates following surgical resection of gastric, lung, colorectal, and esophageal cancers than in non-NCI-designated hospitals [25]. Specific to urology, patients treated by physicians in solo practice receive less-frequent surveillance (a process of care) following a bladder cancer diagnosis than do those whose surgeon is in a group practice [26].

Our results are in keeping with existing literature that describes the influence of provider characteristics and practice environments on the adoption of innovative surgical therapies. For example, prior work identified younger surgeon age, active board certification, urban practice location, group practice affiliation, and a competitive practice setting as important facilitators of general surgeons' adoption of laparoscopic cholecystectomy [27, 28]. Similar findings have been described for surgical treatment in early stage breast cancer [29, 30], as well as urological cancers, such as the use of partial nephrectomy for kidney cancer [31], utilization of continent reconstruction among patients undergoing radical cystectomy for bladder cancer [32], and use of androgen deprivation therapy among patients with localized prostate cancer [22].

This study has several limitations. Because SEER-Medicare data are limited to patients >65 years of age, our findings may not apply to younger patients with kidney cancer. Second, similar to surgery for early-stage breast cancer, clarification of the optimal use of partial nephrectomy and laparoscopy will require a better understanding of patient attitudes and preferences that cannot be assessed using claims data. Third, as we used Medicare claims, we may be underestimating the operative volume of individual surgeons treating patients younger than 65 years. Fourth, we could measure only a limited set of surgeon and practice environment characteristics (most of which are structural in nature); as such, there is a need for future studies that assess the degree to which difficult-to-measure barriers such as technical complexity and/or an absence of adopters in their local communities influence urologists' uptake of these newer surgical therapies.

These limitations notwithstanding, our findings have implications for efforts aimed at facilitating the adoption of partial nephrectomy and laparoscopic radical nephrectomy. As described previously, renewed efforts are needed to better understand barriers to initial and sustained adoption among urologists working in rural environments, small practice settings, and those not affiliated with academic medical centers and/or NCI-designated cancer centers. Although more recently trained urologists were more likely to use laparoscopic radical nephrectomy, our findings counter the notion that uniform adoption will occur naturally as training in this minimally invasive technique becomes more commonplace. Recognizing that social connections and local informational resources facilitate the diffusion of new surgical therapies [27, 33, 34], we see innovative collaborations between urologists, informed by established practice-based surgical research models [35, 36], as representing a potential mechanism for accelerating uniform and equitable adoption of these newer technologies. That being said, the most significant implications from the current study relate to our illustration, more generally, of the power of logic regression as a novel method for discovering interactions in health services and outcomes research. In addition to characteristics of the surgeon and practice environment, others have described multiple contextual factors that influence technology adoption, including, among others, patient demand, professional impact (i.e., financial and social costs), commercial promotion, and magnitude of perceived clinical benefit [37]. As such, methods that allow better characterization and understanding of the complex interplay between these factors will undoubtedly facilitate targeted and efficient interventions to optimize the adoption of both beneficial and potentially harmful new technologies.

## Figures and Tables

**Table 1:** Distribution of patient and tumor characteristics by surgical procedures (1995–2005).

	Total	LPN	LRN	OPN	ORN	*P* value
	*n*	*n* (%)	*n* (%)	*n* (%)	*n* (%)	
	11,918	427 (3.6)	2082 (17.5)	1380 (11.6)	8029 (67.3)	
Age at surgery (years)						0.0001
65–69	3127	131 (4.2)	530 (16.9)	431 (13.8)	2035 (65.1)	
70–74	3423	122 (3.6)	536 (15.7)	426 (12.5)	2339 (68.3)	
75–79	3024	98 (3.2)	579 (19.2)	354 (11.7)	1993 (65.9)	
80–84	1721	59 (3.4)	300 (17.4)	139 (8.1)	1223 (71.1)	
≥85	623	17 (2.7)	137 (22.0)	30 (4.8)	439 (70.5)	
Race/ethnicity						0.0001
Caucasian	9884	344 (3.5)	1757 (17.8)	1158 (11.7)	6625 (67.0)	
African-American	878	33 (3.8)	154 (17.5)	103 (11.7)	588 (67.0)	
Hispanic	719	27 (3.8)	81 (11.3)	70 (9.7)	541 (75.2)	
Other or Unknown	437	23 (5.3)	90 (20.6)	49 (11.2)	275 (62.9)	
Gender						0.0001
Male	6882	274 (3.9)	1134 (16.5)	850 (12.4)	4624 (67.2)	
Female	5036	153 (3.0)	948 (18.8)	530 (10.5)	3405 (67.6)	
Marital status						0.005
Yes	7499	294 (3.9)	1274 (17.0)	901 (12.0)	5030 (67.1)	
No	4419	133 (3.0)	808 (18.3)	479 (10.8)	2999 (67.9)	
Socioeconomic status						0.0001
Low	3808	134 (3.5)	603 (15.8)	424 (11.1)	2647 (69.5)	
Intermediate	3899	135 (3.5)	633 (16.2)	386 (9.9)	2745 (70.4)	
High	4196	158 (3.8)	846 (20.2)	568 (13.5)	2624 (62.5)	
Charlson comorbidity score						0.38
0	6842	241 (3.5)	1186 (17.3)	794 (11.6)	4621 (67.5)	
1	2847	104 (3.7)	512 (18.0)	313 (11.0)	1918 (67.4)	
≥2	1904	74 (3.9)	345 (18.1)	246 (12.9)	1239 (65.1)	
Tumor size (cm)						0.0001
≤4	5188	352 (6.8)	949 (18.3)	1035 (20.0)	2852 (54.9)	
>4	6401	51 (0.8)	1101 (17.2)	286 (4.5)	4963 (77.5)	
Tumor histology						0.0001
Clear cell	10000	301 (3.0)	1682 (16.8)	1042 (10.4)	6975 (69.8)	
Papillary	888	77 (8.7)	200 (22.5)	170 (19.1)	441 (49.7)	
Chromophobe	391	24 (6.1)	107 (27.4)	82 (21.0)	178 (45.5)	
Other	639	25 (3.9)	93 (14.6)	86 (13.5)	435 (68.1)	

**Table 2:** Distribution of surgeon and practice environment characteristics by surgical procedures (1995–2005).

	Total	LPN	LRN	OPN	ORN	*P* value
	*n*	*n* (%)	*n* (%)	*n* (%)	*n* (%)	
	11,918	427 (3.6)	2082 (17.5)	1380 (11.6)	8029 (67.3)	
Surgeon age (years)						0.0001
<40	2553	147 (5.8)	774 (30.3)	271 (10.6)	1361 (53.3)	
40–49	4034	170 (4.2)	728 (18.1)	440 (10.9)	2696 (66.8)	
50–59	3710	85 (2.3)	427 (11.5)	458 (12.4)	2740 (73.9)	
≥60	1621	25 (1.5)	153 (9.4)	211 (13.0)	1232 (76.0)	
Surgeon gender						0.0001
Male	11684	419 (3.6)	2036 (17.4)	1364 (11.7)	7865 (67.3)	
Female	234	8 (3.4)	46 (19.7)	16 (6.8)	164 (70.1)	
Annual nephrectomy volume						0.0001
Bottom 25%	2279	34 (1.5)	209 (9.2)	230 (10.1)	1806 (79.3)	
2nd 25%	3474	77 (2.2)	458 (13.2)	370 (10.7)	2569 (73.9)	
3rd 25%	3141	107 (3.4)	519 (16.5)	320 (10.2)	2195 (69.9)	
Top 25%	3024	209 (6.9)	896 (29.6)	460 (15.2)	1459 (48.3)	
Year of medical school graduation						0.0001
<1960	346	2 (0.6)	9 (2.6)	48 (13.9)	287 (82.9)	
1961–1970	2488	25 (1.0)	176 (7.1)	301 (12.1)	1986 (79.8)	
1971–1980	3568	89 (2.5)	437 (12.3)	403 (11.3)	2639 (73.9)	
1981–1990	3705	166 (4.5)	738 (19.9)	431 (11.6)	2370 (63.9)	
>1991	1811	145 (8.0)	722 (39.9)	197 (10.9)	747 (41.3)	
Practice size						0.0001
Solo or two-person	3200	36 (1.1)	284 (8.9)	279 (8.7)	2601 (81.3)	
Group practice	6619	274 (4.1)	1368 (20.7)	709 (10.7)	4268 (64.5)	
HMO or hospital-based	631	29 (4.6)	100 (15.9)	141 (22.4)	361 (57.2)	
Medical school	484	34 (7.0)	98 (20.3)	125 (25.8)	227 (46.9)	
Other/unclassified	984	54 (5.5)	232 (23.6)	126 (12.8)	572 (58.1)	
Academic affiliation						0.0001
None	4195	88 (2.1)	660 (15.7)	385 (9.2)	3062 (72.9)	
Minor	4408	127 (2.9)	740 (16.8)	420 (9.5)	3121 (70.8)	
Major	3201	207 (6.5)	668 (20.9)	561 (17.5)	1765 (55.1)	
Rural/urban status						0.0001
Urban	11093	412 (3.7)	1992 (17.9)	1318 (11.9)	7371 (66.5)	
Rural	823	15 (1.8)	89 (10.8)	61 (7.4)	658 (79.9)	
Cancer Center Affiliation						0.0001
No	10793	322 (2.9)	1861 (17.2)	1125 (10.4)	7485 (69.4)	
Yes	1096	102 (9.3)	219 (19.9)	252 (22.9)	523 (47.7)	
